# Foundational artificial intelligence models and modern medical practice

**DOI:** 10.1093/bjrai/ubae018

**Published:** 2024-12-18

**Authors:** Alpay Medetalibeyoglu, Yury S Velichko, Eric M Hart, Ulas Bagci

**Affiliations:** Machine and Hybrid Intelligence Lab, Department of Radiology, Northwestern University, Chicago, IL 60611, United States; Department of Radiology, Northwestern University, Chicago, IL 60611, United States; Department of Radiology, Northwestern University, Chicago, IL 60611, United States; Machine and Hybrid Intelligence Lab, Department of Radiology, Northwestern University, Chicago, IL 60611, United States

**Keywords:** artificial intelligence, foundational models, large visual models, data bias, data scarcity and diversity, clinical adoption, precision medicine, modern medicine, large language models, interpretable AI

## Abstract

Our opinion piece pays homage to the evolution of medical practices, tracing back to the era of Hippocrates, through significant historical milestones, and drawing parallels with the principles underpinning foundational artificial intelligence (AI) models. It emphasizes the shared ethos of both domains: a commitment to comprehensive care that values diverse data integration and individualized patient treatment. The excitement surrounding foundation models in medical imaging is understandable. However, a critical and cautious approach is crucial before widespread adoption. By addressing the present 4 major limitations (ie, data bias and generalizability, interpretability of AI models, data scarcity and diversity, and computational resources and infrastructure) and fostering a culture of rigorous research, we can unlock the true potential of these models and revolutionize medical care. This critique (opinion) paper highlights the need for a more measured approach in the field of *foundation AI models* for medicine in general and for medical imaging in particular. It emphasizes the importance of tackling core challenges before rushing toward clinical applications. By focusing on robust methodologies and addressing limitations, researchers can ensure the development of truly impactful and trustworthy models for the betterment of healthcare.

Neither artificial intelligence (AI) nor medical practice is the same as before. Both medical practice and medical AI have undergone dramatic advancements driven by the desire to improve patient care. From stethoscopes to deep learning algorithms, each innovation builds upon the foundation laid by its predecessors. This opinion piece explores the historical moment when these 2 distinct fields, AI and modern medicine, began to converge, marking a significant leap forward with the storm of Foundational AI models. Next, we identify 4 major challenges of Foundational AI models that necessitate a more cautious and critical approach before widespread adoption of them: (1) data bias and generalizability, (2) interpretability of AI models, (3) data scarcity and diversity, and (4) computational resources and infrastructure. Unfortunately, there is a concerning trend in the field: “the proliferation of immature Foundational model application in medicine, particularly in medical imaging.” The emphasis is on rapid publication and novelty, often at the expense of robust methodologies and rigorous validation. This “publish first, ask questions later” approach can impact genuine progress and can undermine trust in the technology.

## From ImageNet to foundational AI: a rapidly changing history of modern AI

The publication of the *ImageNet* classification with deep networks in 2012^1^ marked a critical turning point for modern AI, particularly deep learning, and its significant impact on healthcare. This advancement enabled the use of medical data (text, images, etc.) with ground truth for tasks like clinical decision-making, diagnosis, prognosis, and patient management. Late 2022 witnessed another major leap forward with the introduction of large language models (LLMs) and large visual models (LVMs) to the public.^2^ These models possess broader capabilities unlike specialized AI models designed for specific tasks. Building upon these developments, the recent proposal of *Foundational Models*^2^ holds a great potential to further revolutionize healthcare. By combining LLMs and LVMs, these models (Foundational AI Models) are envisioned as highly trained and adaptable learning tools capable of understanding and working with different medical data, including text reports, images, clinical and pathological data, and even scientific literature. In other words, *Foundational AI models* are large-scale, general-purpose models trained on vast amounts of data that can be adapted to a wide variety of specific tasks. Unlike traditional AI models, which are designed for a single task or domain, foundational models learn broad patterns and representations from diverse data sources, allowing them to generalize across many different applications.^3^ A key characteristic of foundational models is their lack of pre-programmed functionality for a specific task. This allows them to extract and learn broader and complex patterns/features and fundamental relations within the healthcare domain, making them flexible and adaptable. Consequently, they can be fine-tuned and applied to various tasks, ultimately serving as the foundation for building diverse, specialized AI tools within healthcare.

## Roots of computer-aided diagnosis systems and foundational models in clinical practice

Computer-aided diagnosis (CAD) systems are in our life for many decades now, and they primarily focus on detecting abnormalities in medical data to aid diagnosis. They rely on smaller and more specific datasets tailored to a particular disease or imaging modality. Often, they use rule-based, or machine learning algorithms (from pre-deep learning era) trained on labelled data for specific tasks (eg, detecting abnormalities in a specific type of scan). CAD has its roots in clinical practice, closely tied with the “Oslerian” strategy. As an example for disease-specific approach in clinical medicine, Dr William Osler contributed significantly by focusing on specific organs and their diseases. This “Oslerian” method led to categorizing conditions like diabetes into different types, aiding treatments decisions but potentially neglecting individual patient needs.^4^

In addition to disease-specific approaches, medicine has come a long way since the days of Hippocrates, who emphasized treatment tailored to individual needs, much like the *personalized medicine* we strive for today. This focus on the *whole person*, rather than just the disease, has been a consistent thread throughout history, albeit with significant shifts along the way. For an evolved approach as an example, the common approach in healthcare before precision medicine (prior to early 2000s) was “one-size-fits-all” or standardized medicine. This model relied on treating patients based on generalized protocols and population-based averages, rather than individual patient characteristics. The shift toward personalized medicine emphasized the importance of tailoring medical treatments to individual patients based on their genetic, environmental, and lifestyle factors. This personalized approach aligns perfectly with the historical emphasis on treating the *whole person* and represents a significant leap forward from the disease-centric Oslerian approach.^5^ Foundational models have its roots in this strategy as they aim to understand underlying disease processes and predict patient outcome.

Foundational models employ deep learning architectures (more advanced than conventional machine learning strategies) to learn more complex, non-linear relationships within the data, enabling them to identify nuanced patterns and make predictions across various medical tasks. Furthermore, foundational models function as general-purpose models that can be adapted to various medical tasks via fine-tuning or prompting. To do so, these models need to be trained on massive and diverse datasets encompassing various medical data types (patient records, imaging scans, genetic information). Table 1 illustrates the major differences between classical CAD systems and foundational models.

**Table 1.** Major differences between Foundational AI and classical CAD systems.

Feature	Foundational AI	Classical CAD
Data reliance	Massive, diverse datasets	Smaller, specific datasets
Learning approach	Deep learning and generative AI	Rule-based, conventional machine learning
Generality vs specificity	General-purpose	Highly specialized
Interpretability	Challenging	More transparent
Focus	Disease process understanding, outcome prediction	Abnormality detection

## Examples where foundational AI outperforms CAD systems in clinical practice

*Histopathology analysis*: While many CAD systems focus on task-specific model development for pathology image analysis (eg, detecting certain cancer types), foundational AI models provide general-purpose histopathology analysis for multiple tasks, yielding better detection and diagnosis rates.^6^

*Lung nodule malignancy prediction*: CAD systems have moderate-to-high success in predicting lung nodule malignancy in CT scans. Foundational AI models significantly improve these rates by being pre-trained on large amounts of unlabelled data, enabling them to learn more general and robust data representations. They can potentially uncover new biomarkers due to their capacity to integrate larger and multimodal datasets, whereas current CAD systems are limited.^3^

*Multi-modality data integration*: Many CAD systems accept single-modality data and have limited capacity for combining multiple data types. Foundational AI models have the flexibility and power to combine imaging and non-imaging data such as clinical information, lab results, reports, and molecular data, offering a holistic understanding of a patient's condition. For instance, integrating a patient’s CT, MRI, and endoscopic ultrasound imaging with family history and laboratory data can, hypothetically, more accurately assess the risk of developing pancreatic cancer—a feat not achievable with conventional CAD systems.

## What further makes modern medicine and foundational AI models similar?

Both foundational AI models and modern medicine share a crucial understanding: single-faceted approaches have limitations. Just as doctors rely on diverse data points like blood tests, imaging, family history, Foundational AI models excel at processing and analysing varied data types such as genetic, environmental, behavioural, and more.^7^ This comprehensive approach allows both disciplines to paint a more complete picture of the patient or the disease, leading to improved diagnosis, treatment, and even prevention. The similarities extend beyond data analysis. Both Foundational AI models and modern medicine strive for personalized care. By considering individual characteristics and circumstances, they aim to tailor interventions for maximum effectiveness and minimize side effects. This shift toward personalized medicine represents a significant paradigm shift, moving away from the “one-size-fits-all” mentality of the past.

## A case study

Most current AI models in medicine rely on limited, single-type data for risk predictions and diagnoses, making them imperfect yet still valuable. In contrast, foundational AI models go beyond these limitations. They can analyse multiple data types, including genetic, clinical, pathology, imaging, lifestyle, and information from other organs. This enables them to predict cancer risk with greater accuracy, tailor screening approaches to individual needs, and suggest personalized treatment options based on a comprehensive understanding of the patient. Furthermore, unlike the static nature of current models, foundational AI models can track changes over time using multi-modal data. This allows for both adaptive care plans and intervention, as well as facilitating collaborative decision-making through interaction with physicians. Taking an even more holistic approach, these models can incorporate socio-economic and environmental factors into their analysis, providing a more comprehensive picture of individual health.

## The challenges of foundational AI

Foundational AI models hold a great potential, but limitations must be acknowledged.

1. *One major concern is data bias and generalizability:* If training data is skewed, the AI can perpetuate existing healthcare inequalities. Bias can be unwillingly embedded into foundational models due to inherent biases within healthcare data itself (eg, demographics, access to care). Due to their reliance on large datasets, foundational models are especially susceptible to data bias. These biases can lead to unfair or inaccurate results for specific patient populations, hindering model generalizability across diverse settings. For example, lower accuracy in detecting skin cancer with darker skins can be possible due to dominant representation of patient non-colour patient populations in training data. Similarly, foundational AI models for cardiology may show bias if they are trained predominantly on male data, leading to poorer outcomes for female patients, who may present with different symptoms for heart disease. Another example can be that a foundational AI model trained on MRI data from large urban hospitals may fail to generalize well when deployed in smaller, rural clinics that use different equipment or have different patient populations. This could result in misdiagnoses or suboptimal treatment recommendations for those in underserved regions.^7^
2. *Additionally, the interpretability of AI models can be challenging:* Like most other deep learning models, foundational models suffer from a “black box” effect, where the rationale behind their outputs is unclear, and understanding their decision-making process can be difficult. This lack of transparency can raise concerns about accountability and trust. Foundational AI models have an additional difficulty compared to conventional deep learning (DL) systems in medicine: more complex models! Furthermore, interpretation of multi-modal data-based decisions is more challenging than single modality data.
3. *Data scarcity and diversity:* Medical data remains a valuable and often guarded resource due to privacy concerns and institutional silos. This limitation hinders the effectiveness of foundational models, which require massive and diverse datasets. The inherent variability in patient cases poses challenges in data standardization and model generalizability. Moreover, the medical data that is available is often fragmented, with inconsistent formats and varying levels of quality across institutions. This lack of standardized, interoperable datasets makes it difficult to create comprehensive training datasets that can support the broad capabilities of foundational AI models. Data annotation is another challenge; medical data, especially in fields like imaging or genomics, often requires highly specialized, expert-driven labelling, which is time-consuming and costly.
4. *Computational resources and infrastructure:* Training foundational AI models demands significant computational power and specialized hardware such as high-performance graphics processing units (GPUs), tensor processing units (TPUs), or large-scale distributed computing clusters. The sheer size of these models, often containing millions or even billions of parameters, requires extensive processing capacity for both training and fine-tuning on specific medical tasks. This computational burden extends beyond mere training; deploying these models for real-time clinical use also requires significant infrastructure to ensure fast, reliable performance in clinical settings where delays could impact patient care. This creates a barrier for research groups and institutions lacking access to such resources, potentially worsening existing healthcare disparities. Moreover, the environmental impact of running large-scale AI models is becoming an increasing concern. The energy required for training foundational AI models can be vast, contributing to a larger carbon footprint, especially when models are retrained or fine-tuned frequently. This environmental toll adds another layer of complexity, as more sustainable computing methods and energy-efficient algorithms become crucial considerations in scaling AI solutions responsibly.

## Where do we go from here? A future of collaboration

The future of medicine lies in embracing the best of both worlds: the insights of modern medicine and the power of Foundational AI models. This synergy can create a healthcare system that is *truly personalized*, *data-driven*, and *human-centred*. By addressing the challenges of bias, interpretability, and data scarcity and diversity, we can ensure that AI serves as a tool to augment, not replace, the human touch in healthcare.

To mitigate data bias and ensure generalizability, it is imperative to curate diverse and representative training datasets. Collaboration across institutions and regions can facilitate the pooling of data, helping to overcome scarcity and promote inclusivity. Addressing interpretability challenges involves developing methods to elucidate the decision-making processes of complex models, fostering transparency and trust among clinicians and patients. Investing in computational resources and infrastructure is crucial to democratize access to advanced AI tools, preventing the widening of existing disparities. Additionally, establishing rigorous standards for model validation and encouraging a culture of thoroughness over speed in research publications will enhance the reliability of AI applications in medicine.

The future of medicine lies in the synergy between human expertise and advanced AI technologies. By fostering collaboration, promoting ethical practices, and prioritizing patient-centred outcomes, we can harness the full potential of foundational AI models. This integrated approach promises not only to improve individual patient outcomes but also to contribute to a more equitable and effective healthcare system for all. So, let us strive for a future where technology and human expertise work in tandem to create a more holistic and effective approach to medicine.
